# Controlled fabrication of Sn/TiO_2_ nanorods for photoelectrochemical water splitting

**DOI:** 10.1186/1556-276X-8-462

**Published:** 2013-11-05

**Authors:** Bo Sun, Tielin Shi, Zhengchun Peng, Wenjun Sheng, Ting Jiang, Guanglan Liao

**Affiliations:** 1 State Key Laboratory of Digital Manufacturing Equipment and Technology, Huazhong University of Science and Technology, Wuhan 430074, China; 2 Technology Manufacturing Group, Intel Corporation, 2501 NW 229th Ave, Hillsboro, OR 97124, USA

**Keywords:** TiO_2_ nanorods, Sn doping, Photoelectrochemical water splitting

## Abstract

In this work, we investigate the controlled fabrication of Sn-doped TiO_2_ nanorods (Sn/TiO_2_ NRs) for photoelectrochemical water splitting. Sn is incorporated into the rutile TiO_2_ nanorods with Sn/Ti molar ratios ranging from 0% to 3% by a simple solvothermal synthesis method. The obtained Sn/TiO_2_ NRs are single crystalline with a rutile structure. The concentration of Sn in the final nanorods can be well controlled by adjusting the molar ratio of the precursors. Photoelectrochemical experiments are conducted to explore the photocatalytic activity of Sn/TiO_2_ NRs with different doping levels. Under the illumination of solar simulator with the light intensity of 100 mW/cm^2^, our measurements reveal that the photocurrent increases with increasing doping level and reaches the maximum value of 1.01 mA/cm^2^ at −0.4 V versus Ag/AgCl, which corresponds to up to about 50% enhancement compared with the pristine TiO_2_ NRs. The Mott-Schottky plots indicate that incorporation of Sn into TiO_2_ nanorod can significantly increase the charge carrier density, leading to enhanced conductivity of the nanorod. Furthermore, we demonstrate that Sn/TiO_2_ NRs can be a promising candidate for photoanode in photoelectrochemical water splitting because of their excellent chemical stability.

## Background

Growing global energy demand and increasing concern for climate change have aroused the interest in new technologies to harness energy from renewable sources while decreasing dependence on fossil fuels [1,2]. One of the most attractive approaches is to produce hydrogen by solar water splitting, because of the high energy density of hydrogen and zero harmful byproduct after combustion as a fuel [3,4]. Since the first report of the photoelectrochemical water splitting using n-type TiO_2_ in 1972 [5], TiO_2_ has drawn more and more attentions in this field and is regarded as one of the most promising materials as photoanode for solar water splitting, considering its high chemical stability, low cost, and nontoxicity [6,7].

Early efforts in using TiO_2_ material for solar water splitting were mainly focused on the nanoparticle-based thin films for their large surface area-to-volume ratios. However, the high charge carrier recombination and low electron mobility at the grain boundary limit the performance of the films [8,9]. Recently, researches shifted to the one-dimensional nanostructure including nanorods [10-12], nanotubes [13-15], and nanowires [16,17]. Various fabrication processes were developed for the synthesis of TiO_2_ nanorods, nanowires, or nanotubes, such as catalyst-assisted vapor–liquid-solid (VLS) [16], hydrothermal process [10], electrochemical anodization [18,19], etc. However, TiO_2_ is a wide band gap semiconductor, only absorbing UV-light, which suppresses its further applications. Considerable efforts have been devoted to improve the photon absorption and photocatalytic activity of TiO_2_ nanostructures, including synthesizing branched structures [20], exposing its active surface [21], hydrogen annealing process [22,23], and sensitizing with other small band gap semiconductor materials such as PbS [14], CdSe [24], and CuInS [25].

Doping with other elements to tune the band gap of TiO_2_ is another efficient method to improve the photocatalytic activity. N, Ta, Nb, W, and C have been successfully incorporated into TiO_2_ photoanode and been demonstrated with enhanced photoconversion efficiency [26-29]. Besides, the SnO_2_/TiO_2_ composite fibers have also emerged and showed well photocatalytic property [30,31]. Based on these researches, we expect that the incorporation of Sn into TiO_2_ would be an attractive approach since the small lattice mismatch between TiO_2_ and SnO_2_ is beneficial for the structural compatibility and stability. Meanwhile, the doping would significantly increase the density of charge carriers and lead to a substantial enhancement of photocatalytic activity. In this work, we successfully realized the controlled incorporation of Sn into TiO_2_ nanorods by a simple solvothermal synthesis method and investigated the role of Sn doping for enhanced photocatalytic activity in photoelectrochemical water splitting.

## Methods

In our experiments, a transparent conductive fluorine-doped tin oxide (FTO) glass was ultrasonically cleaned in acetone and ethanol for 10 min, respectively, and then rinsed with deionized (DI) water. Twenty-five milliliters DI water was mixed with 25 mL concentrated hydrochloric acid (37%) in a Teflon-lined stainless steel autoclave. The mixture was stirred for several minutes before adding of 0.8 mL tetrabutyl titanate (TBOT). Then, 0–80 μL of Tin tetrachloride (SnCl_4_) solution was added for the synthesis of Sn-doped TiO_2_ nanorods (Sn/TiO_2_ NRs), followed by another several minutes stirring. Subsequently, the clean FTO substrate was placed into the Teflon-liner. The synthesis process was conducted in an electric oven, and the reaction temperature and time were 180°C and 6 h, respectively, for most of the experiments. After that, the autoclave was cooled, and the FTO substrate was taken out and rinsed with DI water. Finally, the sample was annealed at 450°C in quartz tube furnace (Thermo Scientific, Waltham, MA, USA) for 2 h in the air to remove the organic reactant and enhance the crystallization of the nanorods. For the synthesis of pristine TiO_2_ nanorods, the process was all the same, except for the elimination of the Sn precursor. The white nanorods film was detached from the FTO substrate with a blade and then added into ethanol followed by sonication for about 20 min. After that, two drops of the ultrasonically dispersed solution were dropped onto the copper grid and dried by heating in the ambient air for examination. To distinguish the samples with different doping levels, the Sn/TiO_2_ NRs were marked in the form of Sn/TiO_2_-a%, where a% is the molar ratio of SnCl_4_/TBOT.

The morphology and lattice structure of the nanorods were examined by the field-emission scanning electron microscopy (FESEM, JSM-7600 F, JEOL, Akishimashi, Tokyo, Japan) and field-emission transmission electron microscopy (FTEM, Tecnai G2 F30, FEI, Hillsboro, OR, USA). The energy-dispersive X-ray spectroscopy (EDX) combined with FSEM and FTEM was employed to detect the element composition of Sn/TiO_2_ NRs. To further determine the crystal structure and possible phase changes after introducing Sn doping, the crystal structure was examined with X-ray diffraction (XRD, PW3040/60, PANalytical, Almelo, The Netherlands). Moreover, X-ray photoelectron spectroscopy (XPS, VG Multilab 2000 X, Thermo Electron Corp., Waltham, MA, USA) was employed to determine the chemical composition and states of the nanorods. The binding energy of the C 1 s (284.6 eV) was used for the energy calibration, as estimated for an ordinary surface contamination of samples handled under ambient conditions.

To measure the performance of photoelectrochemical (PEC) water splitting, the exposed FTO was covered with a layer of silver paste and connected to Cu wires with solder. The silver paste, solder, edge and some part of the film were sealed with polydimethylsiloxane (PDMS) or epoxy, in which only a well-defined area about 1 cm^2^ of the white film was exposed to the electrolyte. A glass vessel filled with 400 mL 1 M KOH was used as the PEC cell, and a class AAA solar simulator (Oriel 94043A, Newport Corporation, Irvine, CA, USA) with the light intensity of 100 mW/cm^2^ was used as light source. The photocurrent and electrochemical impedance spectra were collected by electrochemical station (AUTOLAB PGSTAT302N, Metrohm Autolab, Utrecht, The Netherlands). Line sweep voltammograms were obtained at the scan rate of 20 mV/S. A Pt slice acting as the counter electrode and a standard Ag/AgCl reference electrode (containing saturated KCl solution) were used for the PEC measurements. The water splitting process in PEC cell was schematically illustrated in (Additional file 1: Figure S1).

## Results and discussion

**Figure 1:**
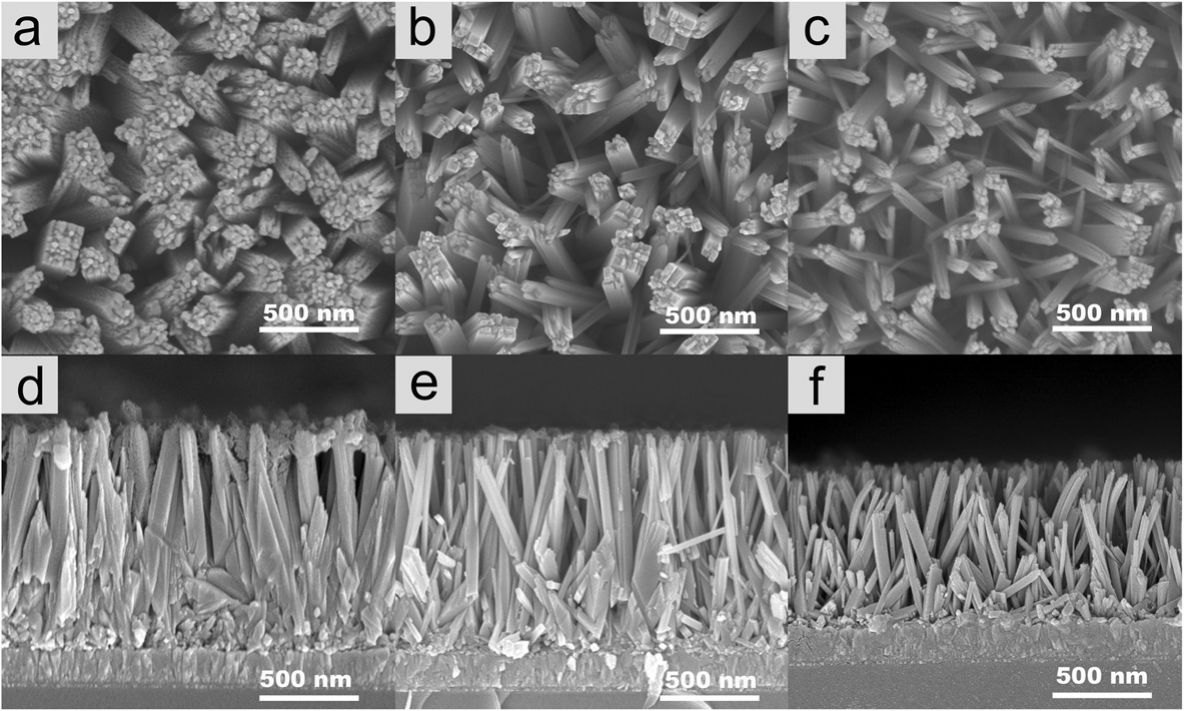
**SEM images of the nanorods synthesized under different conditions. (a)** and **(d)** at 150°C for 18 h; **(b)** and **(e)** at 180°C for 6 h; **(c)** and **(f)** at 180°C for 4 h.

The morphology of the Sn/TiO_2_ nanorods synthesized under different conditions was depicted in Figure 1. Here, Figure 1a,d shows the top view and side view of the nanorods that were synthesized at 150°C for 18 h, Figure 1b,e shows the nanorods synthesized at 180°C for 6 h, and Figure 1c,f shows the nanorods synthesized at 180°C for 4 h, respectively. It reveals that the diameters of the nanorods are about 200, 100, and 80 nm, accordingly, each nanorod consisting of a bundle of thinner nanorods with rectangular top facets. The side view confirms that all the nanorods were grown almost perpendicularly to the FTO substrates, and the average length of the nanorods is 2.1, 2.1, and 1.5 μm, respectively. In order to optimize the surface area-to-volume ratio for PEC water splitting, and enhance the comparability between the nanorods with and without Sn doping, the reaction conditions for median Sn/TiO_2_ nanorods density (Figure 1b,e) were selected for all the remaining experiments in this paper. A wide range of precursor molar ratios (SnCl_4_/TBOT = 0% to 3%) in the initial reactant mixture were used for Sn doping, and almost no noticeable morphology change was observed, except that when the molar ratio reached to 8% the difference turned out to be obvious, as shown in (Additional file 1: Figure S2).

**Figure 2:**
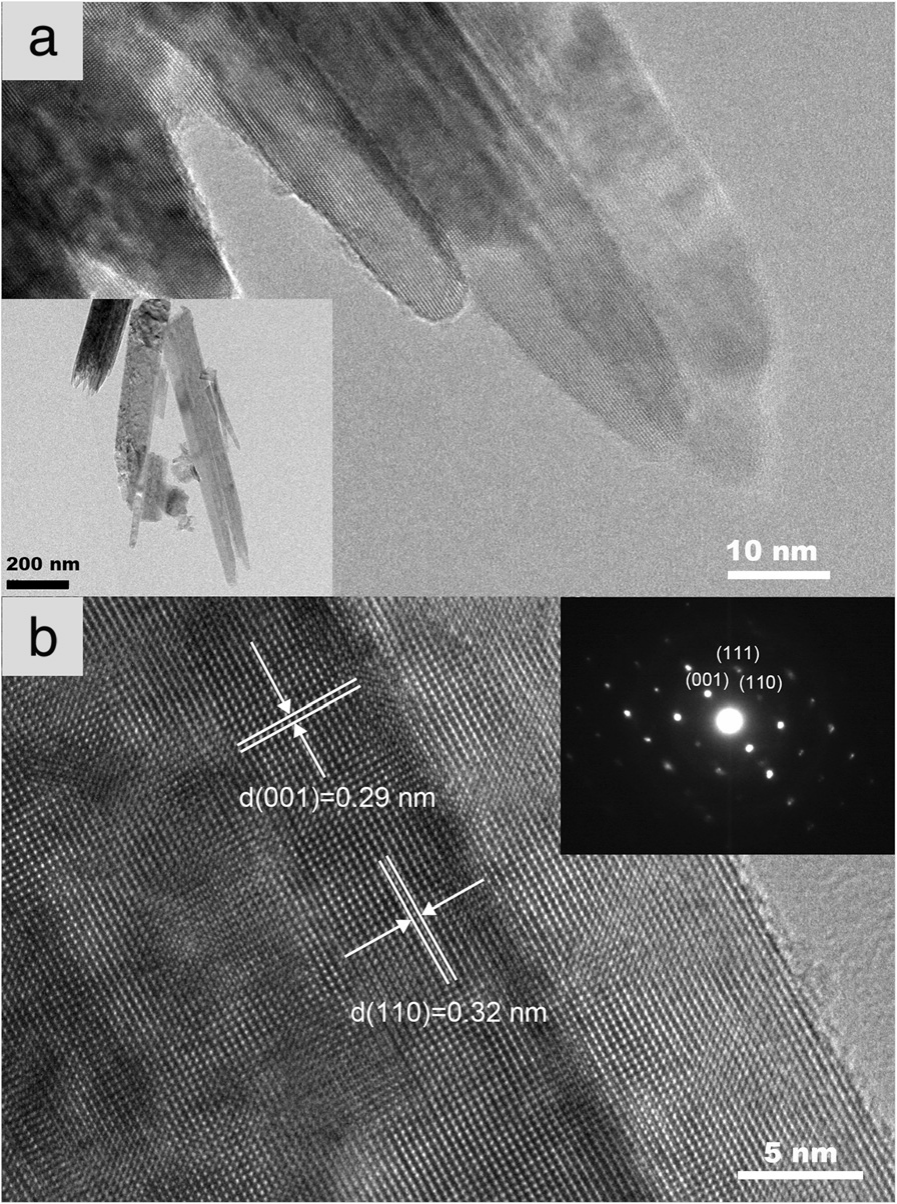
**TEM images and SAED pattern. (a)** TEM image of the tip of a typical Sn/TiO_2_ NR shown in the inset, **(b)** HRTEM image of edge of the nanorod, where the inset is SAED pattern of the nanorod.

Figure 2 displays the TEM images and SAED pattern of a typical Sn/TiO_2_ NR. Although the nanorods detached from the FTO substrate have cracked as shown in the inset of Figure 2a, we can clearly find out that the diameter is about 100 nm, consistent with that measured by SEM in Figure 1b. The image of the nanorod tip confirms that each individual nanorod indeed consists of a bundle of thinner nanorods, with the diameters about 10 to 20 nm. The high-resolution transmission electron microscopy (HRTEM) image collected from the edge of the nanorods reveals that the typical Sn/TiO_2_ NR has a single crystalline structure with the interplanar spacings of 0.32 nm and 0.29 nm, in accordance with the d-spacings of (110) and (001) planes of rutile TiO_2_, respectively. These results indicate that the Sn/TiO_2_ NR grows along the <001 > direction. The sharp SAED pattern as shown in the inset of Figure 2b further confirms that the Sn/TiO_2_ NR is a single crystalline rutile structure.

**Figure 3:**
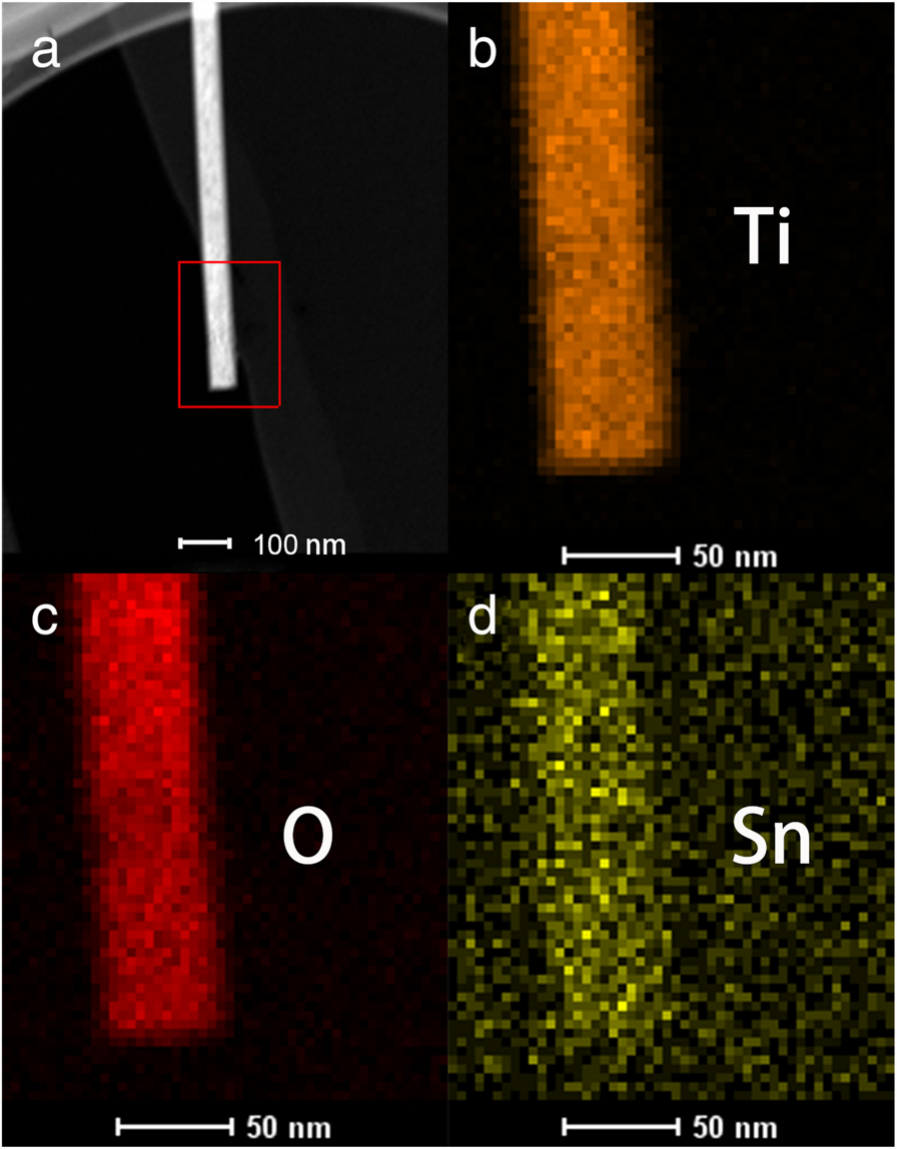
**HAADF scanning TEM image and elemental mappings of a Sn/TiO**_**2**_**-0.5% NR. (a)** HAADF scanning TEM image, **(b), (c),** and **(d)** is the elemental mappings of Ti, O, and Sn, collected from the nanorod within the rectangular region marked in **(a)**.

Figure 3 illustrates the element composition of a representative Sn/TiO_2_-0.5% NR obtained by EDX. Figure 3a shows the high-angle annular dark-field (HAADF) scanning TEM image of the nanorod, while Figure 3b,c,d are elemental mappings of Ti, O, and Sn, respectively, collected from the nanorod within the rectangular region marked in Figure 3a. Although this percentage of Sn/Ti has approached to the detection limit of EDX and some background noise have kicked in, we can find that Sn atoms have been incorporated over the entire TiO_2_ nanorod obviously in Figure 3d. Besides, the Sn/Ti ratios of all the detected samples are close to the SnCl_4_/TBOT molar ratios as shown in (Additional file 1: Figure S3).

**Figure 4:**
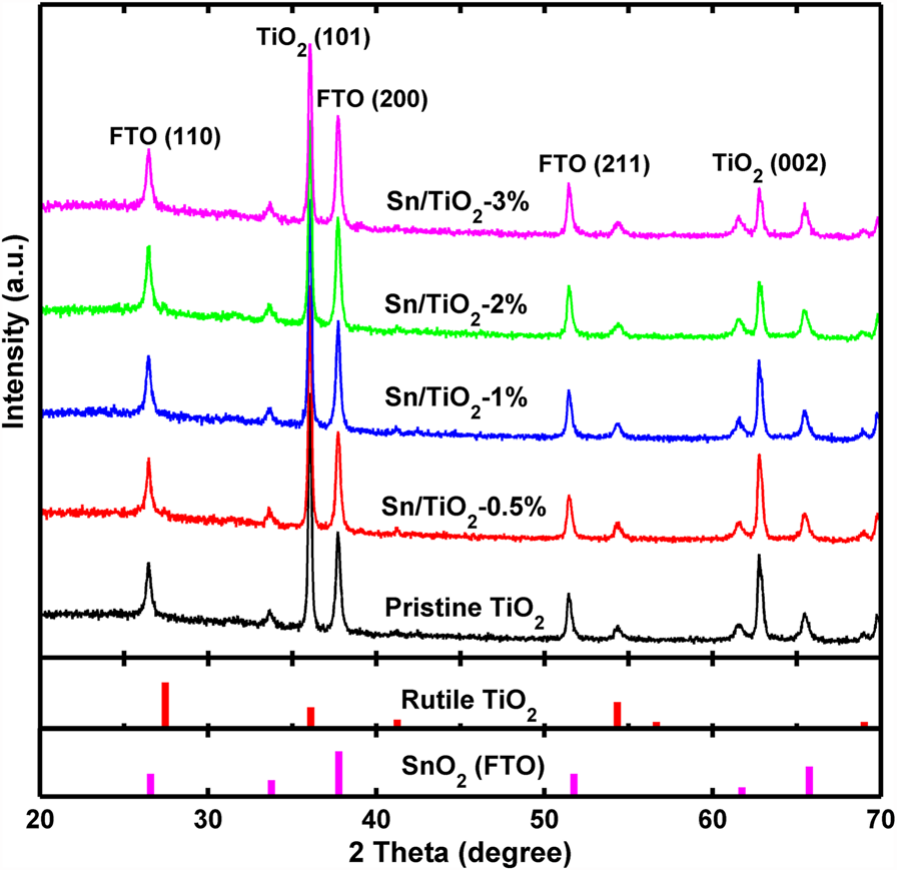
**XRD patterns of pristine TiO**_**2 **_**NRs and Sn/TiO**_**2 **_**NRs synthesized with different precursor molar ratio.** The reference spectra (JCPDS No. 21–1276 and No. 46–1088) were plotted for comparison.

To further determine the crystal structure and possible phase changes after Sn doping, we collected the XRD spectra from pristine TiO_2_ NRs and Sn/TiO_2_ NRs synthesized with different precursor molar ratio, as shown in Figure 4, in which the typical diffraction peaks of the patterns have been marked. It confirms that the Sn/TiO_2_ NRs have a tetragonal rutile TiO_2_ crystal structure (JCPDS No. 21–1276), which is the same as the pristine TiO_2_ NRs. Even for the highly doped sample (Sn/TiO_2_-3%), there is no obvious change in diffraction peaks. We infer that the Sn atoms just replace Ti atoms in some spots without destroy the rutile TiO_2_ crystal structure as schematically illustrated in (Additional file 1: Figure S4). Noteworthy is that the relative intensity of (002) peaks seems to decrease as the doping level exceed 2%. This change may result from the fact that the perpendicularity of the nanorods to the substrate has reduced, as demonstrated in (Additional file 1: Figure S2).

**Figure 5:**
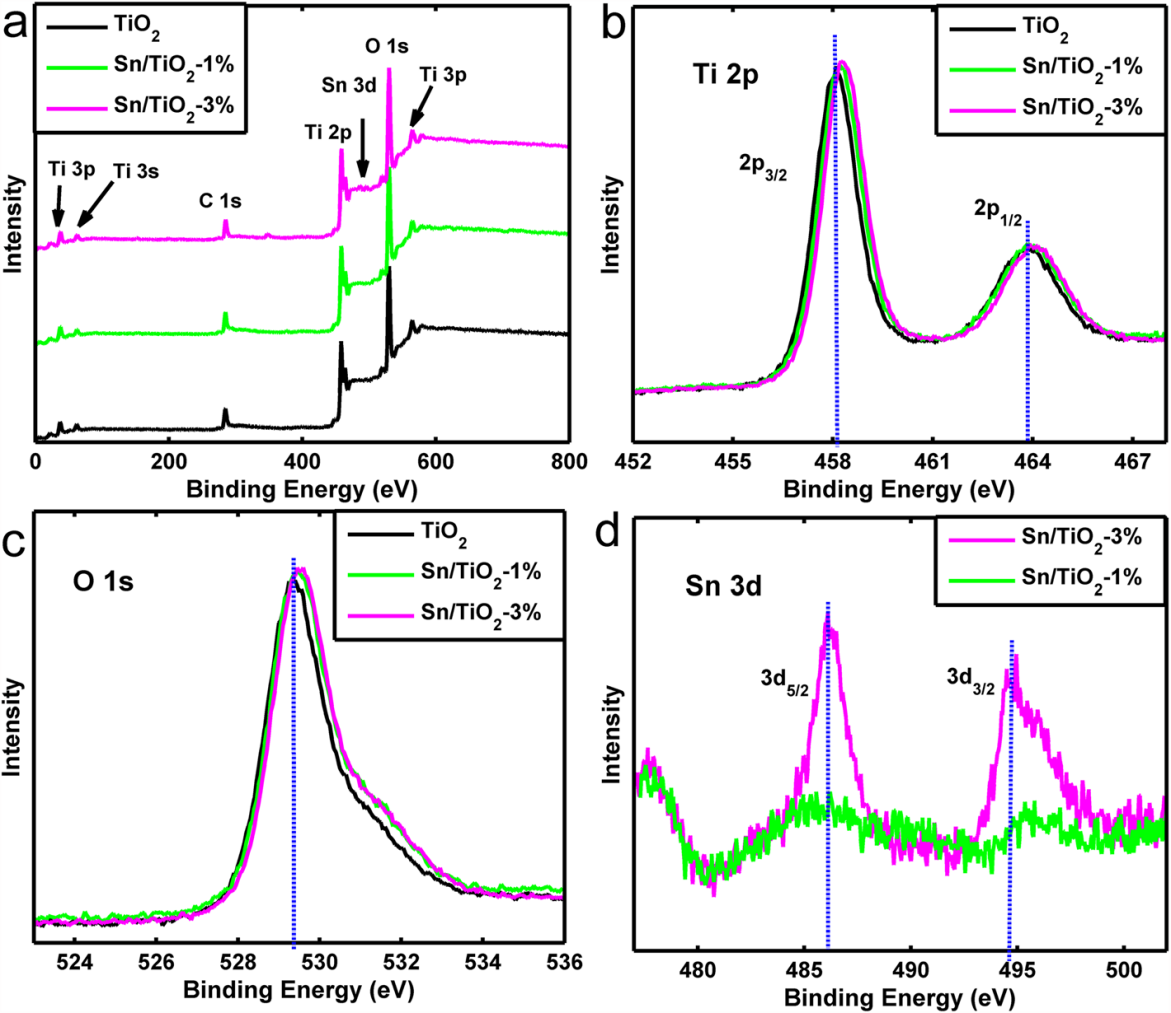
**XPS survey spectra. (a)** Low-resolution XPS survey spectra of the pristine TiO_2_ NRs and Sn/TiO_2_ NRs with different Sn doping, **(b)** Ti 2p XPS spectra, **(c)** O 1 s XPS spectra; **(d)** Sn 3d XPS spectra.

To investigate the changes of the surface composition and chemical states of TiO_2_ NRs after introducing Sn doping, the XPS spectra collected from the pristine TiO_2_ NRs and two representative Sn/TiO_2_ NRs samples with initial SnCl_4_/TBOT molar ratio of 1% and 3% are compared in Figure 5a. The XPS peaks of the TiO_2_ NRs (with or without Sn doping) at about 458.1 and 463.9 eV correspond to Ti 2p_3/2_ and Ti 2p_1/2_ (Figure 5b), and the XPS peak at about 529.4 eV corresponds to O 1 s state (Figure 5c), respectively. In Figure 5d, the two peaks of the spectra collected from Sn/TiO_2_-3% NRs at about 486.2 and 494.8 eV correspond to Sn 3d_5/2_ and Sn 3d_3/2_, which confirms that the main dopant is Sn^4+^. Compared to the pristine TiO_2_ NRs, the Ti and O peaks of Sn/TiO_2_ NRs show a small positive shift (as shown in Figure 5b,c), which suggests a certain electron drain from the Ti^4+^ in the oxide matrix due to the presence of Sn^4+^, considering their difference in electronegativity (Sn = 1.96 vs. Ti = 1.54) [32]. This further confirms that the Sn dopant is indeed mixed into TiO_2_ NRs at the atomic level, agreeing well with the XRD results as shown in Figure 4. Besides, quantitative analysis of the spectra reveals that the Sn/Ti molar ratio is about 1.2% for Sn/TiO_2_-1% NRs and 3.2% for Sn/TiO_2_-3% NRs, respectively.

**Figure 6:**
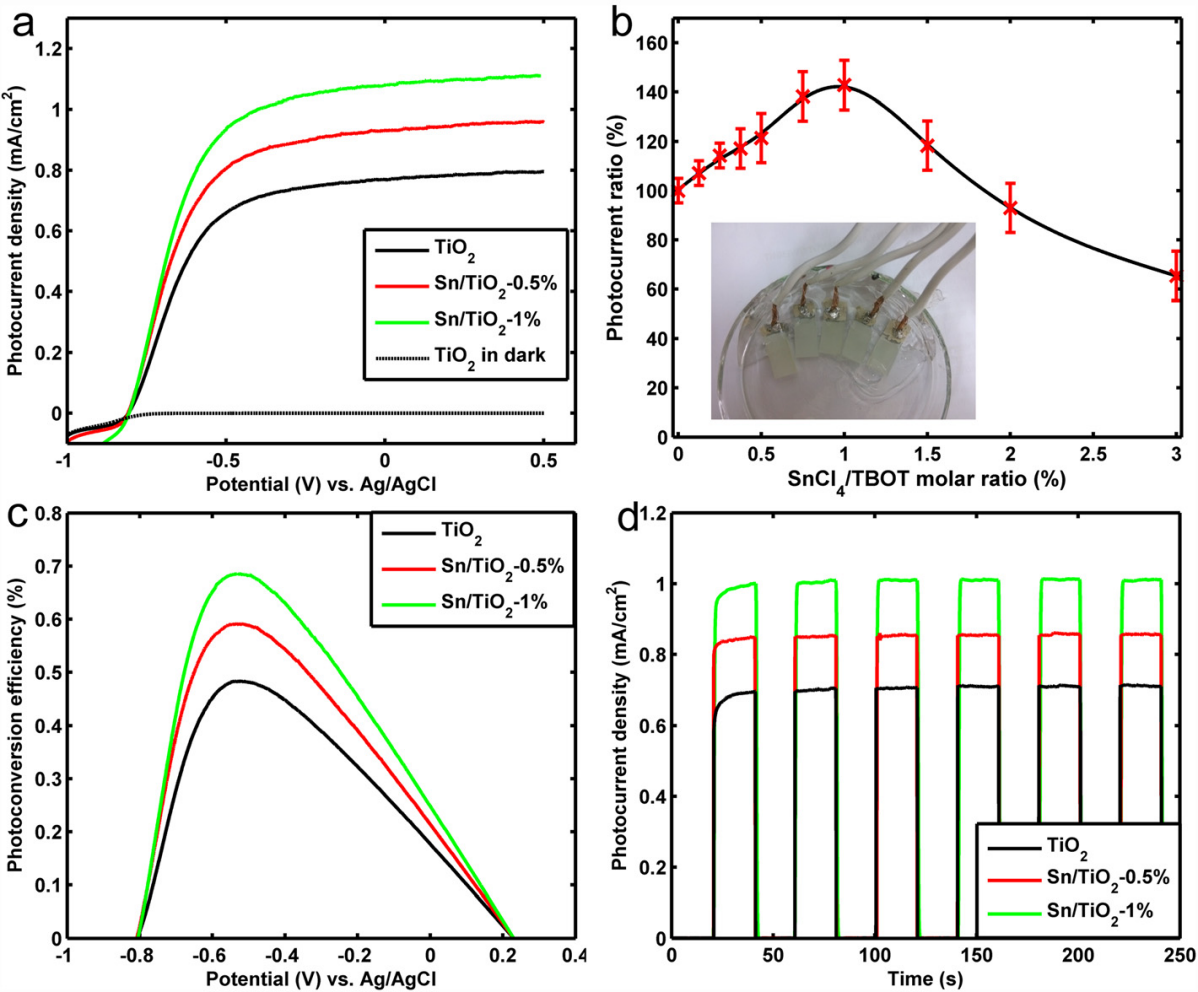
**Photocatalytic properties of the nanorods. (a)** Line sweep voltammograms measured from pristine TiO_2_ NRs (black), Sn/TiO_2_-0.5% NRs (red), and Sn/TiO_2_-1% NRs (green). The current of the pristine TiO_2_ NRs in dark is plotted for comparison. **(b)** Photocurrent density ratios between Sn/TiO_2_ NRs and pristine TiO_2_ NRs photoanodes measured at −0.4 V versus Ag/AgCl, and the inset is optical photo of a few typical packaged samples. **(c)** Photoconversion efficiency of the three samples as a function of applied voltage versus Ag/AgCl. **(d)** Time-dependent photocurrent density of the three samples at repeated on/off cycles of illumination from the solar simulator.

Next, the photocatalytic activities of the Sn/TiO_2_ NRs with different Sn doping levels for PEC water splitting are discussed. Figure 6a displays the line sweep voltammograms measured from pristine TiO_2_ NRs (black), Sn/TiO_2_-0.5% NRs (red), and Sn/TiO_2_-1% NRs (green), and the current of the pristine TiO_2_ NRs in dark is plotted in black dotted line for comparison. The photocurrent density of pristine TiO_2_ is 0.71 and 0.77 mA/cm^2^ at the potential of −0.4 and 0 V versus Ag/AgCl, while the value increases to 0.85 and 0.93 mA/cm^2^ for the Sn/TiO_2_-0.5% NRs and reaches 1.01 and 1.08 mA/cm^2^ for the Sn/TiO_2_-1% NRs. To further explore the effect of Sn doping on the photocatalytic activity, the photocurrent measurements were conducted for a series of samples synthesized with the precursor molar ratio from 0% to 3%. The photocurrent density ratios between Sn/TiO_2_ NRs and pristine TiO_2_ NRs photoanodes measured at −0.4 V versus Ag/AgCl are depicted in Figure 6b, where the inset is the optical image of the packaged Sn/TiO_2_ NR photoanodes. The results reveal that the photocurrent first increases as the doping level rises and reaches the max value of 142 ± 10% at precursor molar ratio of 1%, which corresponds to up to about 50% enhancement compared to pristine TiO_2_ NRs sample, and then decreases gradually and drops to a value even lower than that of a pristine nanorods.

To analyze the efficiency of Sn/TiO_2_ NRs for PEC water splitting quantitatively, the photoconversion efficiency is calculated as follow [33]:

$$\eta =J\left(1.23-V\right)/P,$$

where *J* is the photocurrent density at the measured potential, *V* is the applied voltage versus reversible hydrogen electrode (RHE), and *P* is the power intensity of 100 mW/cm^2^ (AM 1.5G). The RHE potential can be converted from the Ag/AgCl reference electrode [23]

$$\mathrm{Voltage}\;\left(\mathrm{RHE}\right)=\mathrm{Voltage}\;\left(\mathrm{Ag}/\mathrm{AgCI}\right)+1.0V.$$

As shown in Figure 6c, the pristine TiO_2_ NRs achieve the efficiency of 0.48%, while the Sn/TiO_2_-0.5% NRs and Sn/TiO_2_-1% NRs achieve the efficiencies of 0.59% and 0.69% at about −0.53 V versus Ag/AgCl, about 23% and 44% enhancement, respectively. The photocatalytic properties of TiO_2_ and Sn/TiO_2_-1% nanorods with different morphology were depicted in (Additional file 1: Figure S5), which further supports our choice of the reaction conditions for median nanorods density. These results suggest that appropriate incorporation of Sn atoms can significantly enhance the photocatalytic activity of TiO_2_ NRs and lead to substantial increase of the photocurrent density and photoconversion efficiency.

The time-dependent measurements also have been carried out on the three samples, as shown in Figure 6d. With repeated on/off cycles of illumination from the solar simulator, the three samples display highly stable photocurrent densities of 0.71, 0.86 and 1.01 mA/cm^2^ at −0.4 V versus Ag/AgCl, respectively. These measurements have been repeated in several months, and there is no noticeable change happened. This indicates that the Sn/TiO_2_ NRs possess highly chemical and structural stability for PEC water splitting, which is another critical factor to evaluate their potentials as the photoanode material.

**Figure 7:**
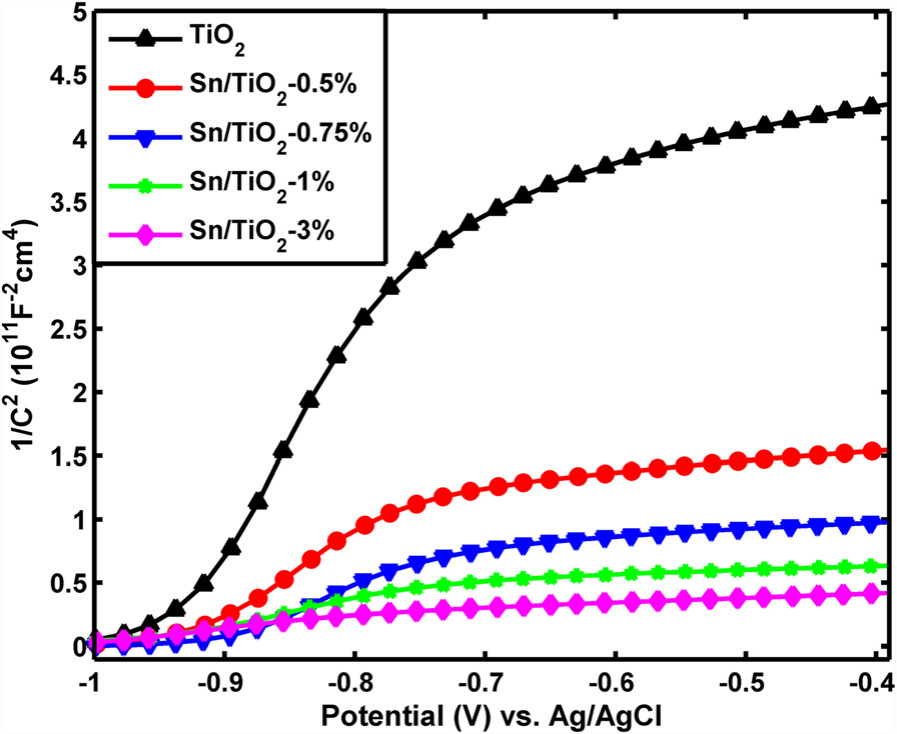
**Mott-Schottky plots for the pristine TiO**_**2 **_**NRs and Sn/TiO**_**2 **_**NRs with different doping levels.** The data were collected at a frequency of 5 kHz in the dark.

To investigate the role of Sn doping on the enhanced photocatalytic activity, especially for its influence on the electronic properties of TiO_2_ NRs, we have conducted electrochemical impedance measurement on the pristine TiO_2_ and Sn/TiO_2_ NRs with different doping levels at the frequency of 5 kHz in dark as shown in Figure 7. All the samples measured show a positive slope in the Mott-Schottky plots, as expected for TiO_2_ which is a well-known n-type semiconductor. Importantly, the Sn-doped TiO_2_ NRs samples show substantially smaller slopes than that of the pristine TiO_2_ NRs, suggesting a significantly increase of charge carrier densities. Furthermore, the slope decreased gradually as the precursor molar ratio increased from 0.5% to 3%, which confirms the role of Sn doping on increasing the charge carrier density. The carrier densities of these nanorods can be calculated from the slopes of Mott-Schottky plots using the equation [23]

$$N_{d}=\left(\frac{2}{e_{0}\epsilon \epsilon_{0}}\right){\left[\frac{d\left(\frac{1}{C^{2}}\right)}{\mathrm{dV}}\right]}^{-1},$$

where *N*_*d*_ is the charge carrier density, *e*_0_ is the electron charge, *ϵ* is the dielectric constant of TiO_2_ (*ϵ* = 170) [23], and *ϵ*_0_ is the permittivity of vacuum. The calculated charge carrier densities of the pristine TiO_2_, Sn/TiO_2_-1% and Sn/TiO_2_-3% NRs are 5.5 × 10^17^, 7.85 × 10^18^, and 1.25 × 10^19^ carries/cm^3^, respectively. We note that the Mott-Schottky method is derived based on a flat electrode model and may have errors in determining the accurate value of charge carrier density of the Sn/TiO_2_ NRs, since we use the planar area instead of the effective surface area for calculation [34]. Nevertheless, the qualitative comparison of the carrier density of these samples is valid, given that there is almost no difference between the morphology of pristine TiO_2_ and Sn/TiO_2_ NRs.

As oxygen vacancy serving as electron donor has been accepted generally as the main cause for the n-type conductivity of TiO_2_[35], we expect that the incorporation of Sn atoms may lead to the increase of oxygen vacancy which is responsible for the enhanced photocatalytic activity. Besides, other reported effects may also be at work. For instance, the formation of mixed-cation composition (Sn_*x*_Ti_1−*x*_O_2_) at the interface and associated modulation of electronic properties may facilitate the exciton generation and separation [30]. The potential difference of TiO_2_ and SnO_2_ may promote the photoelectron migration from TiO_2_ to SnO_2_ conducting band with decreasing combination, allowing both of the photogenerated electrons and holes to participate in the overall photocatalytic reaction [31]. However, the photocurrent of Sn/TiO_2_-3% NRs is lower to the pristine TiO_2_. This may be rationalized as the overly high Sn doping level upshifting the TiO_2_ band gap and creating much more interfaces, which substantially reduces the light absorption efficiency and impedes the photogenerated charge separation.

## Conclusions

In summary, we have successfully realized the controlled incorporation of Sn into TiO_2_ NRs to enhance the photocatalytic activity for PEC water splitting. Sn concentration is well controlled by adjusting the precursor molar ratio. We studied the crystal structure of the obtained Sn/TiO_2_ NRs, which is the same as the pristine TiO_2_ NRs. The PEC measurements reveal that the photocurrent reaches the maximum value of 1.01 mA/cm^2^ at −0.4 V versus Ag/AgCl with a Sn/Ti molar ratio of about 1%, which corresponds to up to about 50% enhancement compared to the pristine TiO_2_ NRs. The Mott-Schottky plots indicate that the incorporation of Sn into TiO_2_ NRs can significantly increase the charge carrier density, hence improving the conductivity of TiO_2_ NRs and leading to the increase of photocurrent. Besides, the Sn/TiO_2_ NRs exhibit excellent chemical stability which further promotes them to be a promising candidate for photoanode in photoelectrochemical water splitting devices. With the enhanced conductivity, we believe the Sn/TiO_2_ NRs can also serves as substitution for pure TiO_2_ structures in other optoelectronic applications including photocatalysis, photodetectors, solar cells, etc.

## Competing interests

The authors declare that they have no competing interests.

## Authors’ contributions

BS carried out experimental work, analyzed the data, and prepared the manuscript. TLS participated in the studies and supervised the research work. ZCP improved the manuscript. WJS and TJ participated in the experimental work. GLL participated in the studies, improved the manuscript, and supervised the research work. All authors read and approved the final manuscript.

## Supplementary Material

Additional file 1: Figure S1 Schematic illustration of the water splitting process in PEC cell. **Figure S2.** SEM images of the Sn/TiO_2_ NRs with different doping levels, (a) Sn/TiO_2_-0.5% NRs, (b) Sn/TiO_2_-8% NRs. **Figure S3.** EDX spectra measured from a series of Sn/TiO_2_ NRs, with initial SnCl_4_/TBOT ratio range from 0.5% to 8%, (a) 0.5%, (b) 1%, (c) 1.5%, (d) 2%, (e) 3%, (f) 8%, the marked values in the spectra are detected Sn/Ti ratio. **Figure S4.** A supercell for modeling the crystal structure of the Sn/TiO_2_ NRs. **Figure S5.** The photocatalytic properties of TiO_2_ and Sn/TiO_2_ nanorods with different morphology, (a) photoconversion density, (b) photoconversion efficiency. Click here for file
