# Supplementary material for: Controlled fabrication of Sn/TiO2 nanorods for photoelectrochemical water splitting
Source: Nanoscale Res Lett. 2013 Nov 5;8(1):462. doi: 10.1186/1556-276X-8-462 (PMC4228341; doi:10.1186/1556-276X-8-462)
Supplement: Additional file 1: Figure S1 — Schematic illustration of the water splitting process in PEC cell. Figure S2. SEM images of the Sn/TiO2 NRs with different doping levels, (a) Sn/TiO2-0.5% NRs, (b) Sn/TiO2-8% NRs. Figure S3. EDX spectra measured from a series of Sn/TiO2 NRs, with initial SnCl4/TBOT ratio range from 0.5% to 8%, (a) 0.5%, (b) 1%, (c) 1.5%, (d) 2%, (e) 3%, (f) 8%, the marked values in the spectra are detected Sn/Ti ratio. Figure S4. A supercell for modeling the crystal structure of the Sn/TiO2 NRs. Figure S5. The photocatalytic properties of TiO2 and Sn/TiO2 nanorods with different morphology, (a) photoconversion density, (b) photoconversion efficiency. [file 1556-276X-8-462-S1.pdf]

Supporting information for:

Controlled fabrication of Sn/TiO_2 nanorods for photoelectrochemical water splitting

Bo Sun,[1] Tielin Shi,[1] Zhengchun Peng,[2] Wenjun Sheng,[1] Ting Jiang[1] and Guanglan Liao[1] *

1 State Key Laboratory of Digital Manufacturing Equipment and Technology, Huazhong University of Science and Technology, Wuhan 430074, China

2 Technology Manufacturing Group, Intel Corporation, 2501 NW 229th Ave, Hillsboro, OR 97124, USA

*corresponding author, tel: +86-27-87793103, fax: +86-27-87793103, e-mail: guanglan.liao@hust.edu.cn

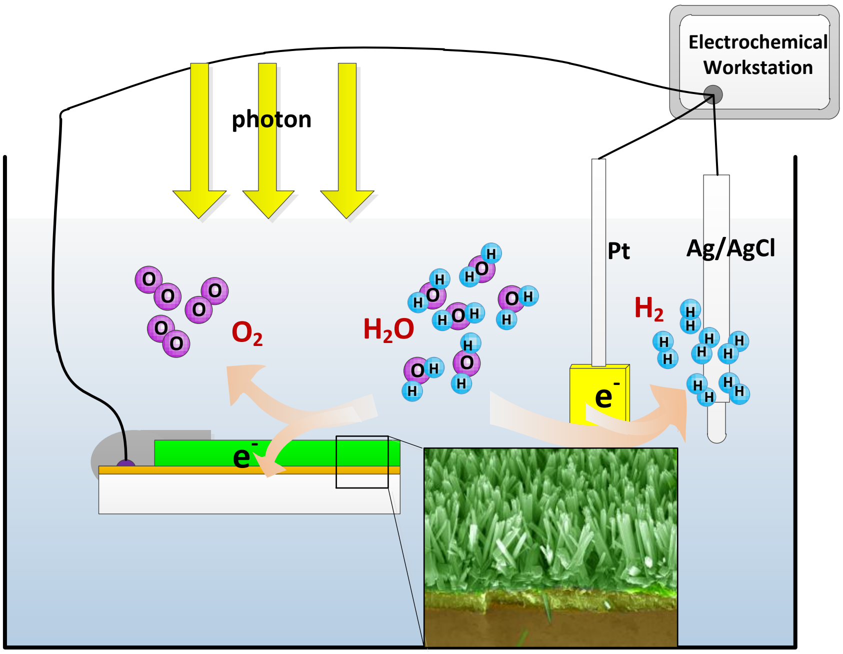

Figure S1: Schematic illustration of the water splitting process in PEC cell.

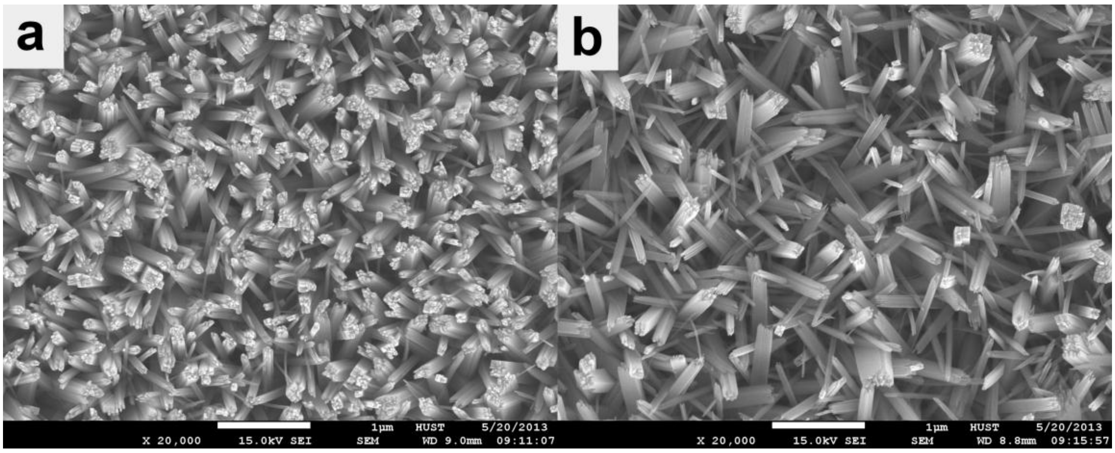

Figure S2: SEM images of the Sn/TiO_2 NRs with different doping levels, (a) Sn/TiO_2-0.5% NRs, (b) Sn/TiO_2-8% NRs.

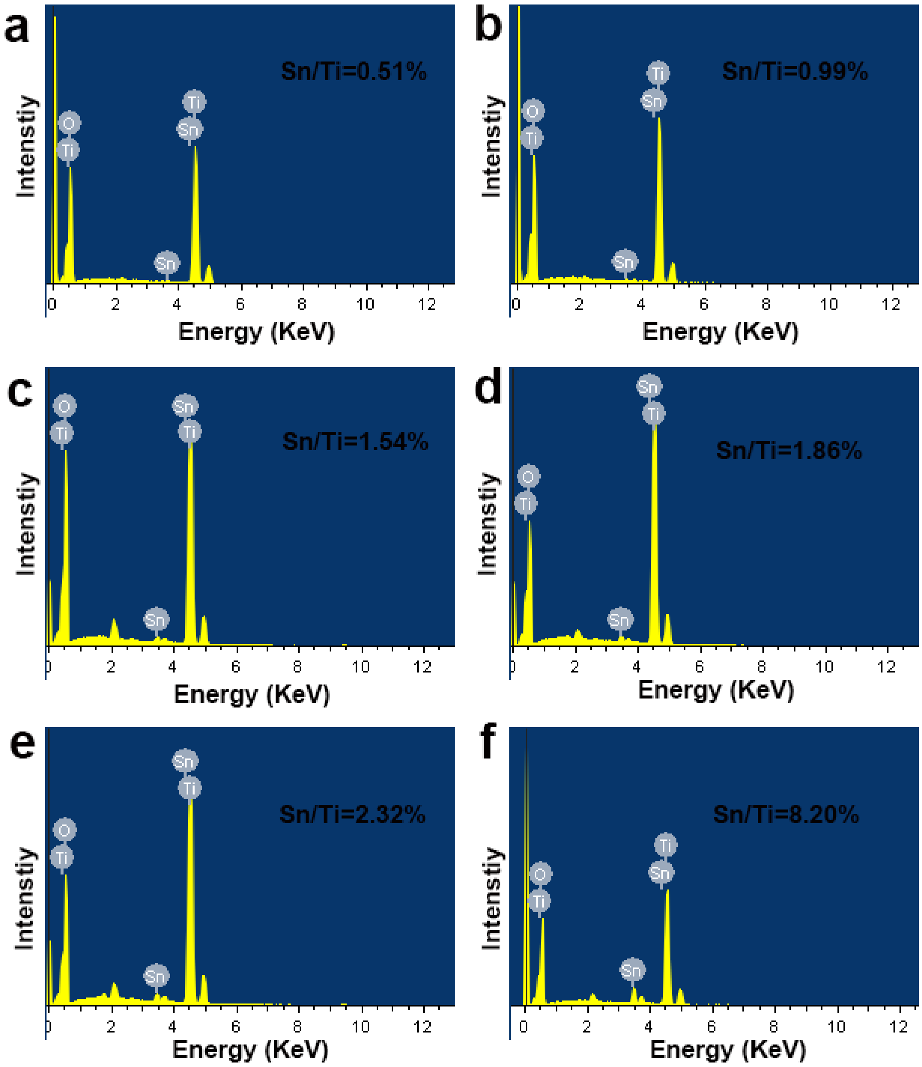

Figure S3: EDX spectra measured from a series of Sn/TiO_2 NRs, with initial $SnCl_4$ /TBOT ratio range from 0.5% to 8%, (a) 0.5%, (b) 1%, (c) 1.5%, (d) 2%, (e) 3%, (f) 8%, the marked values in the spectra are detected Sn/Ti ratio.

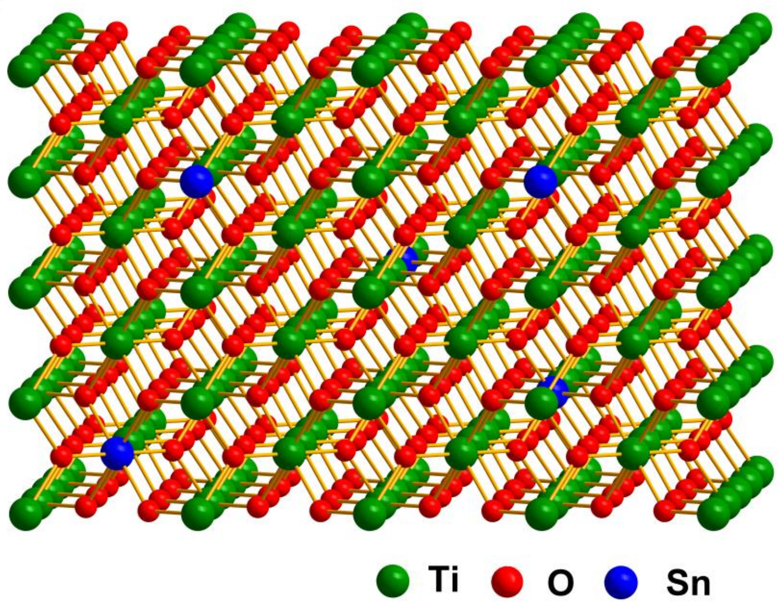

Figure S4: A supercell for modeling the crystal structure of the Sn/TiO_2 NRs.

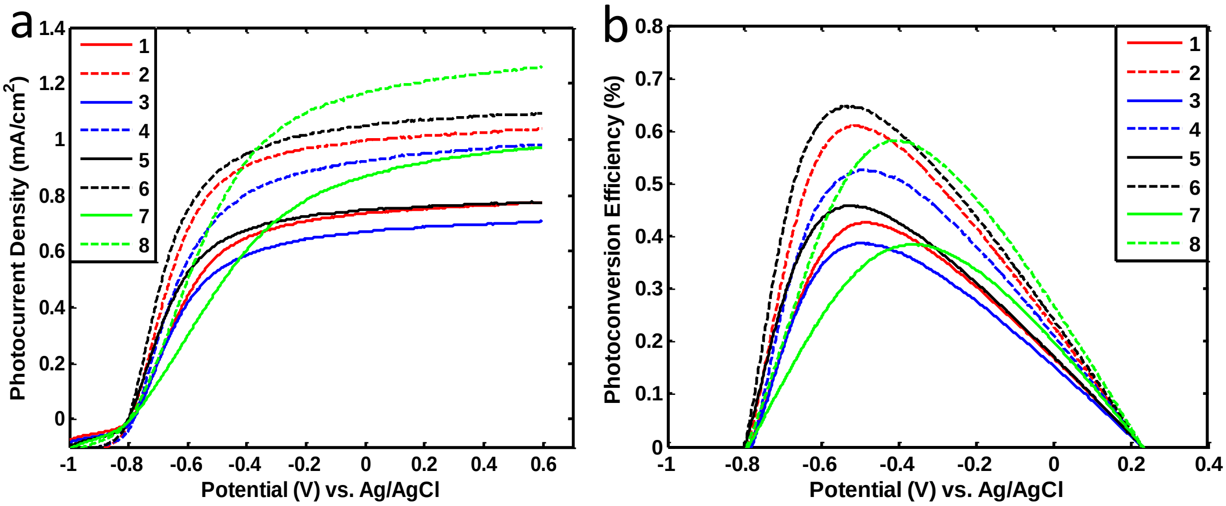

Figure S5: The photocatalytic properties of TiO_2 and Sn/TiO_2 nanorods with different morphology, (a) photoconversion density, (b) photoconversion efficiency. (1) TiO_2 nanorods synthesized under 150℃ for 18 hours, (2) Sn/TiO_2-1% nanorods synthesized under 150℃ for 18 hours, (3) TiO_2 nanorods synthesized under 180℃ for 4 hours, (4) Sn/TiO_2-1% nanorods synthesized under 180℃ for 4 hours, (5) TiO_2 nanorods synthesized under 180℃ for 6 hours, (6) Sn/TiO_2-1% nanorods synthesized under 180℃ for 6 hours, (7) TiO_2 nanorods synthesized under 180℃ for 6 hours for twice, (8) Sn/TiO_2-1% nanorods synthesized under 180℃ for 6 hours for twice.
